# Systemic Immune-Inflammation Index in Relation to Diabetes Markers in Saudi Adults: A Retrospective Study

**DOI:** 10.3390/medicina60030442

**Published:** 2024-03-07

**Authors:** Ghadeer S. Aljuraiban, Fahad J. Alharbi, Ali O. Aljohi, Abdullah Z. Almeshari, Masoud N. Alotaibi, Salem S. AlShammari, Sara Al-Musharaf, Madhawi M. Aldhwayan, Manal Abudawood

**Affiliations:** 1 Department of Community Health Sciences, College of Applied Medical Sciences, King Saud University, Riyadh 11362, Saudi Arabia; salmosharruf@ksu.edu.sa (S.A.-M.); maldhwayan@ksu.edu.sa (M.M.A.); 2 Central Military Laboratory & Blood Bank, Prince Sultan Military Medical City, Riyadh 11159, Saudi Arabia; fjharbi@psmmc.med.sa (F.J.A.); aaljohi@psmmc.med.sa (A.O.A.); ameshari@psmmc.med.sa (A.Z.A.); 3 Deputyship of Research Chairs, Deanship of Scientific Research, King Saud University, Riyadh 11362, Saudi Arabia; alomasoud@ksu.edu.sa; 4 Department of Clinical Laboratory Sciences, College of Applied Medical Sciences, King Saud University, Riyadh 11362, Saudi Arabia; salshammari1@ksu.edu.sa (S.S.A.); mabudawood@ksu.edu.sa (M.A.); 5 Center of Excellence in Biotechnology Research, King Saud University, Riyadh 11451, Saudi Arabia

**Keywords:** inflammatory markers, systemic immune-inflammation index, diabetes, diabetes markers, fasting blood glucose, HbA1c

## Abstract

*Background and objectives*: Low-grade inflammation is associated with metabolic disturbances like diabetes. The systemic immune-inflammation index (SII) has been proposed as a predictive tool to identify individuals at a greater risk of diabetes. This study aims to examine the association between SII and diabetes markers. *Method and materials*: We used retrospective data from a large cohort of adults (*n* = 3895) aged ≥18 in Saudi Arabia. The SII was calculated, and the markers of diabetes such as fasting blood glucose (FBG), insulin, and hemoglobin A1c (HbA1c) were included. *Results*: Across the quartiles of SII, FBG, insulin, and HbA1c were significantly higher in adults with higher compared to lower SII (*p* < 0.0001, *p* = 0.04, *p* < 0.0001, respectively). A two SD higher FBG was significantly associated with an SII difference of 47.7 (95% CI: (15.5, 91.9)). In subgroup analysis, this relationship prevailed in normal-weight participants and among those with normoglycemia and prediabetes but was attenuated in participants with diabetes. The association also prevailed in separate analyses for males and females but was stronger among females. Linear regression models showed no significant association between insulin, HbA1c, and SII. *Conclusions*: SII was associated with the markers of diabetes. The utility of SII for predicting diabetes can be confirmed with prospective cohort studies.

## 1. Introduction

Diabetes is considered a worldwide health crisis, with around 537 million adults aged 20–79 years living with diabetes in 2021, a number that is projected to rise in all parts of the world over the coming years [1]. The impact of type 2 diabetes is also astounding, affecting 462 million people of all ages in 2017, the equivalent of 6.28% of the global population [2]. Diabetes impacts individual, economic, and social well-being. In addition to the numerous physical complications related to poor glycemic control (e.g., coronary heart disease, stroke, peripheral vascular disease, end-stage renal disease, neuropathy, retinopathy, and lower-extremity amputation), diabetes also greatly affects quality of life, with adverse consequences on professional productivity, mental health, family life, and others, along with extensive economic and healthcare costs [3,4,5].

Saudi Arabia is not exempt from this burden as it has one of the highest rates of type 2 diabetes in the Middle East [6]. The pooled prevalence of type 2 diabetes in Saudi Arabia ranges from 16.4% for all ages from studies published between 2000 and 2020 [7] to 28% among adults from studies published between 2016 and 2022 [8]. The direct healthcare costs of those diagnosed with diabetes in Saudi Arabia were estimated at SAR 17 billion in 2014 [9]. However, many people remain undiagnosed, and the direct cost would have been as high as SAR 27 billion if the undiagnosed were included among those being treated [9].

It has been posited that the increasing rates of diabetes can, in part, be attributed to low-grade chronic inflammation and the inducement of insulin resistance by it [10]. Novel prognostic scores, like the systemic immune-inflammation index (SII), can help identify patients at risk of poor outcomes for whom the predictive capability of traditional clinicopathologic signs is inefficient [11]. The SII is a prognostic score based on platelet, neutrophil, and lymphocyte counts that represents the status of systemic inflammation, in which a higher score is usually indicative of poorer prognoses [11]. Perhaps, because SII incorporates more features of the inflammatory response, using three individual parameters [11], some studies have demonstrated that SII has a stronger relationship with health outcomes compared to other inflammatory scores (e.g., neutrophil-to-lymphocyte ratio, platelet-to-lymphocyte ratio, and monocyte-to-lymphocyte ratio), indicating its potential to be used for the early warning of personal inflammatory response [12,13,14]. Further, like other inflammatory scores, SII presents a non-invasive, low-cost option, drawing from biochemical markers frequently collected for routine blood work [11]. The SII was initially developed as a prognostic indicator of outcomes for patients with hepatocellular carcinoma after surgery and showed powerful and promising results [11]. Since its inception, SII has demonstrated its potential utility as a prognostic or predictive score for patients with various cancers [15,16,17], cardiovascular diseases [18], COVID-19 [19,20], and other diseases [21,22].

Recent examinations of data from the National Health and Nutrition Examination Survey (NHANES) 2017–2020 [23] and 2011–2018 [24] from the US found that SII was positively associated with the risk of diabetes. Establishing SII as a predictive tool to identify individuals at a greater risk of diabetes presents an affordable and routine method to enable early interventions and stratify risk. Cohort studies can further support the findings from the cross-sectional NHANES studies [24]. This research aims to examine the association between SII and diabetes markers using retrospective data from a large cohort of adults in Saudi Arabia.

## 2. Materials and Methods

### 2.1. Study Design

For this retrospective study, data from Prince Sultan Military Medical City were analyzed. The registry contains population and laboratory data for a total of 4732 patients, collected between 2022 and 2023. Data were collected for both inpatients and outpatients from all departments during this time. Following the exclusion of 837 people with missing SII and diabetes marker data and those using insulin, 3895 participants were ultimately included in the study. The Institutional Review Board (IRB) at the Prince Sultan Military Medical City approved the study (IRB number: E-2115).

### 2.2. Diabetes Markers

Type 2 diabetes, prediabetes, and normoglycemia diagnoses were all confirmed by a physician and were available from patient hospital records. According to hospital protocol, a patient was considered to have normoglycemia when they had a fasting blood glucose (FBG) (8 h overnight) less than 100 mg/dL, or a 2 h plasma glucose tolerance test of less than 140 mg/dL, or a hemoglobin A1c (HbA1c), the average blood glucose level over the last 2–3 months, of 5.6% (38 mmol/mol) or less.

Prediabetes, when blood glucose level is higher than normal but not high enough to be considered diabetes, was reported when FBG was 100 mg/dL to 125 mg/dL, or when a 2 h plasma glucose tolerance test was 140 mg/dL to 199 mg/dL, or HbA1c was between 5.7% to 6.4% (39 mmol/mol to 46 mmol/mol).

Diabetes was diagnosed either by a FBG level of 126 mg/dL or higher, or a 2 h plasma glucose tolerance test of 200 mg/dL or higher, or a HbA1c of 6.5% (48 mmol/mol) or higher, confirmed by a repeated test on a different day.

### 2.3. Blood Samples

Biochemical data were available for each participant. Blood samples were collected routinely for patients according to protocol and transported to the central laboratory. Quality assurance and control of all laboratory equipment were carried out regularly.

FBG and insulin levels were measured utilizing a Cobas-8000 autoanalyzer (Roche Diagnostics, Switzerland) using the enzymatic reference method with hexokinase (Roche, Cas No: 8717). HbA1c level was measured using a Cobas-513 autoanalyzer (Roche Diagnostics, Rotkreuz, Switzerland) using Tina-Quant HbA1c Gen. 3 (Roche, Cas No: 29162). Complete blood count (CBC), including counts of platelets, neutrophils, lymphocytes, and white blood cells, was measured utilizing Sysmex XN analyzer (Sysmex, Kobe, Japan). To calculate SII, the following formula was applied [11]:

$$SII=\frac{Platelet\;count\;\left(10^{9}/L\right)\times Neutrophil\;count\;\left(10^{9}/L\right)}{Lymphocyte\;count\;\left(10^{9}/L\right)}$$

### 2.4. Anthropometric Data

Blood pressure was routinely measured and recorded by trained staff following the protocol using Omron HEM 705-CP (OMRON Corp., Kyoto, Japan). If a patient had multiple blood pressure measurements, the average measurement was used. Hypertension was defined as systolic blood pressure (SBP) ≥ 130 mmHg and/or diastolic blood pressure (DBP) ≥ 80 mmHg [25].

Weight and height were recorded using standard hospital measures using a weighing scale and a portable stadiometer (Marsden H226, Marsden Weighing Group, South Yorkshire, UK). Patients were instructed to wear lightweight clothing for their weight measurement. Body mass index (BMI) was calculated by dividing body weight (kg) by height (m^2^). BMI was classified as normal if BMI ≤ 25.0 kg/m^2^, overweight if BMI > 25.0 and ≤30.0 kg/m^2^, and obese if BMI > 30.0 kg/m^2^.

### 2.5. Statistical Analysis

SAS version 9.3 by SAS Institute in Cary, NC, USA, was used for all statistical analyses. Baseline characteristics were presented as means and standard deviations (SD) for continuous variables and as frequencies for categorical variables. We used linear regression (PROC GLM) to calculate age- and sex-adjusted means of baseline characteristics stratified by quartiles of SII (Q1 < 267.4, Q2 ≥ 267.4 and <400.7, Q3 ≥ 400.7 and <588.5, Q4 ≥ 588.5).

Multivariate linear regression models adjusted for variables that could potentially confound the association between SII and diabetes were used to identify associations between SII and higher increments (by 2 SD) of FBG (68.8 mg/dL), insulin (31.2 pmol/L), and HbA1c (3.5%). Model 1 was adjusted for age (y) and sex (male/female). Model 2 was additionally adjusted for white blood cell count, platelet count, BMI, and the presence of hypertension (hypertensive/non-hypertensive). Using interaction terms, the potential effect modification by sex, BMI, and age was examined.

Stratified analyses by sex, BMI group, presence of hypertension (hypertensive/non-hypertensive), diabetes status (normoglycemia, prediabetes, diabetes), and age (categorized according to the Saudi General Authority of Statistics [26] into ≤24.0 y, 24.1–54.0 y, 54.1–64.0 y, and ≥64.1 y) was performed. Findings with a *p*-value of <0.05 were considered statistically significant.

## 3. Results

### 3.1. Baseline Characteristics

The sample included 3895 participants (1585 males and 2310 females) with an average age (SD) of 48.6 (±18.6) years (Table 1). About 32.9% had normoglycemia, 42.1% had prediabetes, and 25.0% had diabetes. In total participants, 76.0% were normal weight, 10.0% were overweight, and 14% were obese. The average BMI was 29.6 (±8.1) kg/m^2^. In total participants, mean SII was 500.5 (±217.6), FBG was 52.4 (±34.4) mg/dL, insulin was 14.8 (±11.6) pmol/L, and HbA1c was 6.7 (1.8)%.

Across quartiles of SII, FBG was significantly higher in those with higher (Q4 ≥ 588.5) compared to lower (Q1 < 267.4) SII, [62.1 (95% CI: 58.7, 65.4) vs. 42.0 (95% CI: 37.5, 46.6 mg/dL *p* < 0.0001)]. Similarly, insulin and HbA1c were higher in those with higher compared to lower SII [6.6 (95% CI: −5.2, 18.4) vs. 9.8 (95% CI: −6.1, 25.8, pmol/L, *p* = 0.04)] and [6.6 (95% CI: 6.5, 6.7) vs. 6.9 (95% CI: 6.8, 6.9%, *p* < 0.0001)], respectively (Table 2).

### 3.2. Relationship between Systemic Immune-Inflammation Index and Fasting Blood Glucose

Overall, two SD higher FBG was significantly associated with an SII difference of 47.7 (95% CI: (15.5, 91.9)) (Model 2; Table 3).

Subgroup analysis revealed that the association between FBG and SII prevailed in those with normoglycemia and prediabetes, with SII differences of 79.1 (95% CI: 33.1, 125.2) and 47.8 (21.1, 74.4), respectively. However, the association was attenuated among those with diabetes.

Across BMI subgroups, the association between FBG and SII prevailed in normal-weight participants 30.3 (95% CI: 13.0, 47.6), and was not significant among those with overweight and obesity.

The association also prevailed in separate analyses for male and female participants, with a stronger relationship among females compared to males [37.2 (95% CI: 12.6, 61.7) vs. 24.2 (95% CI: 3.1, 45.4)] for females and males, respectively].

In age subgroups, the association prevailed in older participants (>65 y) only: 51.7 (95% CI: 15.8, 87.5) (Table 3).

### 3.3. Relationship between Systemic Immune-Inflammation Index, Insulin, and HbA1c

There was no significant association between insulin, HbA1c, and SII, overall or in any of the subgroups (Table 4).

## 4. Discussion

This study represents the first assessment of the association between SII and diabetes markers in a cohort of Saudi Arabian adults. When examining the cohort as a whole, we found a positive relationship between SII and the markers of diabetes (i.e., FBG, HbA1c, insulin). This connection prevailed for some subgroup analyses. Specifically, there was a positive association between HbA1c and SII for females and between FBG and SII for females, adults > 65 years, and people with normal weight. Furthermore, the relationship with FBG was strongest for people with prediabetes compared to those with normoglycemia or diabetes.

Our results align with previous findings that identified a positive relationship between SII and diabetes or its complications [23,24,27,28,29,30]. In addition to the examinations of SII and diabetes prevalence from NHANES data [23,24], other studies have demonstrated an association between SII and various diabetes-related complications and mortality [27,28,29,30]. For instance, a high SII was strongly associated with the development of diabetic macular edema [28], diabetic kidney disease [29], osteomyelitis [30], and all-cause and cardiovascular mortality among those with diabetes [27]. The relationship between SII and diabetes or its complications is unsurprising given some of the mechanisms underlying the development of diabetes and its complications [31]. Inflammation and the immune response play important roles in the pathophysiology and progression of diabetes [31]. Specifically, chronic low-grade inflammation contributes to organ dysfunction and tissue damage, which can promote insulin resistance and impaired insulin secretion [31].

The present study found sex differences when examining subgroups, which aligns with some of the findings from cross-sectional studies [23,24]. Stratified analyses in the study by Liu and colleagues found significant relationships between SII and type 2 diabetes for both males and females and a significant *p*-value for the interaction effect of sex [24]. On the other hand, Nie et al. found that overall, sex had no significant impact on the relationship between SII and diabetes. In stratified analyses, the nature of the relationship for males and females presented differently [23]. The association between SII and diabetes, when examined among females, followed an inverted U-shaped curve, like the overall trend they saw, but when examined among males, it followed a more linear trend [23]. Variations in results between studies may be due to methodological differences. For instance, the outcome definition for Liu et al. [24] included measures of HbA1c, FBG, diabetes medication use, and diagnoses by a medical professional, similar to the present study. For Nie et al. [23], the definition included only diagnoses by a medical professional.

In general, it is plausible that the variables of sex, age, and BMI interact with the relationship between SII and diabetes. Each of these variables has a noted relationship with diabetes and/or inflammatory response and, as such, would be adjusted for in statistical models [1,23,24,32]. In their meta-analysis, Alwadeai et al. found that people in Saudi Arabia over 40 years old were at a greater risk of type 2 diabetes, and the risk for women and those that were overweight or obese (compared to normal weight) also trended higher (non-significantly) [8]. Age is a noted factor in the development of diabetes, and the number of people living with diabetes in the world is predicted to continue with increasing age [1]. Likewise, sex and gender differences are noted in immune responses, with females showing a stronger response than males, potentially because of hormonal, genetic, or social differences [32]. Research from China attempting to define reference intervals for SII and other inflammatory scores found that SII scores did not differ by gender or age; however, this research included a cohort of healthy adults from another country, and therefore, the results may not be generalizable to the current study [33]. Interestingly, our study found that the relationship between SII and FBG prevailed among individuals with normal weight but not among those that were overweight or obese in subgroup analyses. It is possible that the higher risk of diabetes and higher SII with increasing BMI levels attenuates the relationship between the two, leaving the strongest association among those with normal weight. While Nie et al. found normal weight (but not overweight or obesity) significantly correlated with SII, the interaction tests showed BMI as a variable overall did not impact the relationship between SII and diabetes [23]. Liu et al. found that BMI may affect the relationship between SII and diabetes in interaction tests. Future research can be conducted to further understand this phenomenon.

The strength of the relationship between SII and fasting blood glucose for those with prediabetes compared to people with and without diabetes may result from the fluctuating levels of inflammation and immune response that vary with the progression and development of type 2 diabetes as well as by the marker examined [34]. Grossman and colleagues examined the immune and inflammatory response of people with normoglycemia, prediabetes, and diabetes [34]. They found that the levels of several inflammatory and immune biomarkers (i.e., white blood cells, granulocytes (primarily neutrophils), monocytes, interleukin (IL)-1 receptor antagonist, IL-18, and fibrinogen) increased when examining the progression from normoglycemia to prediabetes to diabetes, they found an increase in lymphocytes and c-reactive protein from normoglycemia to prediabetes, and an increase in neopterin concentrations from prediabetes to diabetes [34]. It is apparent from this study that various measures of inflammation and immune response change at different rates depending on the stage of diabetes progression [34]. It is possible that SII, with the strongest relationship among those with prediabetes, could be used as a predictive marker of subclinical disease to identify individuals at risk of diabetes. The early identification of risk for diabetes can avert microvascular and macrovascular complications associated with hyperglycemia and reduce or delay the progression to type 2 diabetes [35]. Using available and effective therapies to forestall type 2 diabetes and its complications can be more efficient than treating complications after they develop [35]. Using measures of inflammation to predict type 2 diabetes has been performed in the past [36,37]. For instance, low-grade inflammation measured with acute-phase markers has been shown to predict type 2 diabetes incidence in some groups in the US [36,37].

The capability of SII for the purpose of predicting type 2 diabetes can be explicitly examined with future research efforts using a prospective cohort design. Prospective cohorts can allow for the assessment of temporal sequence to establish if those with high SII develop diabetes [38]. The versatility of SII for predicting poor health outcomes has been explored for other conditions and has been identified as an important biomarker because it provides substantial information for predictive models, especially for cancers [39]. A recent review by Islam et al. [39] concludes that SII and other indices are affordable and dependable and can help clinicians make critical decisions. However, their ability to predict a specific health condition as a standalone metric may be limited [39]. Research efforts in the future can explore such applications for diabetes.

A strength of this study was the sample size. We used a large and diverse registry of adults in Saudi Arabia that allowed us to examine the relationship between SII and diabetes markers overall and provided the means to analyze the relationship among various subgroups. We had information on insulin use, which we used to exclude those on insulin from the analysis. Additionally, diabetes diagnoses were based on physician diagnoses rather than self-report, which is subject to self-report bias, a type of measurement error [40]. A common limitation of retrospective analyses is that all relevant information is unlikely to be collected since the cohort was prepared originally for a different purpose [24]. As such, some lifestyle data, for instance, diet and physical activity, were unavailable. While these aspects have been shown to relate to diabetes risk [41], the studies by Nie et al. [23] and Liu et al. [24] showed that adjustment for these and other factors did not negate the positive association between SII and diabetes. Other variables not included in the cohort data (e.g., monocyte counts) precluded the investigation of other systemic indices, such as the systemic inflammatory response index (SIRI) and the aggregate index of systemic inflammation (AISI). Furthermore, the generalizability of these results to other cohorts may not be applicable. For instance, the cohort participants were patients of the Prince Sultan Military Medical City and, therefore, would not be generalizable to a healthy population. However, using a cohort of patients enabled us to compare people at various stages in the development of diabetes and allowed us to incorporate clinical comorbidities (e.g., BMI) into our analyses. Additional considerations for generalizability should reflect geographical location and cultural and environmental factors.

## 5. Conclusions

Overall, our study found a positive relationship between SII and the markers of diabetes, and this relationship persisted for several subgroups upon examination. This study is the first, to our knowledge, to assess this relationship using retrospective cohort data and presents the first conducted among adults in Saudi Arabia. These results and the utility of SII for predicting diabetes risk can be confirmed with prospective cohort studies in the future.

## Figures and Tables

**Table 1:** Baseline characteristics, *n* = 3895 ^a^.

Variable	Mean or %	
Age (y)	48.6	(±18.6)
Age groups (y)		
≤24	11.4%	
25–54	48.4%	
55–64	20.8%	
≥65	19.4%	
Male	40.7%	
BMI (kg/m^2^)	29.6	(±18.0)
Normal weight	76.0%	
Overweight	10.0%	
Obese	14.0%	
SBP (mmHg)	126.3	(±19.5)
DBP (mmHg)	74.4	(±11.4)
Hypertension	14.0%	
Neutrophil 10^2^/L	3.7	(±1.8)
Platelet 10^9^/L	298.9	(±84.1)
Lymphocyte 10^9^/L	2.5	(±0.9)
White Blood Cells 10^9^/L	7.0	(±2.4)
SII	500.5	(±217.6)
FBG (mg/dL)	52.4	(±34.4)
Insulin (pmol/L)	14.8	(±11.6)
HbA1c%	6.7	(±1.8)
Normoglycemia	32.9%	
Prediabetes	42.1%	
Diabetes	25.0%	

^a^ Data are presented as mean (±SD) or %. BMI: body mass index; DBP: diastolic blood pressure; FBG: fasting blood glucose; HbA1c: hemoglobin A1c; SBP: systolic blood pressure; and SII: systemic immune-inflammation index.

**Table 2:** Characteristics stratified by quartiles of systemic immune-inflammation index, *n* = 3895 ^a^.

	SII				
	Q1 (<267.4)	Q2 (≥267.4 and <400.7)	Q3 (≥400.7 and <588.5)	Q4 (≥588.5)	*p* Value
	Mean (95% CI)	Mean (95% CI)	Mean (95% CI)	Mean (95% CI)	
*n*	973	974	974	974	
SII (median)	200.9	332.3	477.4	785.0	
Age (y)	49.6 (48.8, 50.5)	48.3 (47.2, 49.5)	47.4 (46.2, 48.6)	48.1 (46.9, 49.3)	0.01
BMI (kg/m^2^)	29.6 (28.2, 31.1)	31.3 (29.1, 33.4)	29.2 (27.1, 31.2)	28.4 (26.5, 30.3)	0.28
SBP (mmHg)	126.9 (125.8, 128.1)	125.7 (124.2, 127.2)	127.1 (125.6, 128.6)	125.3 (123.9, 126.7)	0.17
DBP (mmHg)	74.9 (74.2, 75.6)	74.3 (73.4, 75.3)	74.1 (73.2, 75.1)	73.8 (72.9, 74.7)	0.31
Neutrophil 10^9^/L	2.1 (2.0, 2.2)	3.1 (3.0, 3.2)	3.9 (3.9, 4.0)	5.5 (5.4, 5.5)	<0.0001
Platelet 10^9^/L	260.1 (255.4, 264.7)	285.8 (281.1, 290.5)	309.2 (304.5, 313.9)	341.3 (336.6, 346.0)	<0.0001
Lymphocyte 10^9^/L	2.8 (2.8, 2.9)	2.6 (2.6, 2.7)	2.5 (2.4, 2.5)	2.1 (2.0, 2.1)	<0.0001
White Blood Cells 10^9^/L	5.7 (5.6, 5.9)	6.5 (6.4, 6.6)	7.3 (7.1, 7.4)	8.3 (8.2, 8.5)	<0.0001
FBG (mg/dL)	42.0 (37.5, 46.6)	51.6 (47.1, 56.1)	45.7 (41.1, 50.2)	62.1 (58.7, 65.4)	<0.0001
Insulin (pmol/L)	6.6 (−5.2, 18.4)	14.2 (−14.9, 43.3)	42.9 (24.2, 61.6)	9.8 (−6.1, 25.8)	0.04
HbA1c%	6.6 (6.5, 6.7)	6.6 (6.5, 6.7)	6.6 (6.5, 6.7)	6.9 (6.8, 6.9)	<0.0001

^a^ Linear regression (PROC GLM) presented as mean (95% CI) adjusted for age and sex unless otherwise specified. BMI: body mass index; DBP: diastolic blood pressure; FBG: fasting blood glucose; HbA1c: hemoglobin A1c; SBP: systolic blood pressure; and SII: systemic immune-inflammation index.

**Table 3:** Estimated mean difference in SII associated with 2 SD higher FBG, overall and by subgroups, *n* = 3895.

	SII	R^2^	*p* Value
	Mean Difference (95% CI)		
Fasting blood glucose			
Model 1 ^a^	34.2 (18.1, 50.2)	0.041	<0.0001
Model 2 ^b^	47.7 (3.5, 91.9)	0.065	<0.0001
*Subgroup analysis*			
Normoglycemia	79.1 (33.1, 125.2)	0.198	0.0008
Prediabetes	47.8 (21.1, 74.4)	0.105	0.0005
Diabetes	26.5 (−55.0, 2.0)	0.078	0.07
Normal weight	30.3 (13.0, 47.6)	0.063	0.0006
Overweight	45.9 (−109.9, 18.0)	0.137	0.16
Obesity	39.6 (−90.5, 11.3)	0.072	0.13
Female	37.2 (12.6, 61.7)	0.238	0.003
Male	24.2 (3.1, 45.4)	0.056	0.03
Age groups (y)			
≤24	27.7 (−114.3, 59.0)	0.088	0.53
25–54	15.4 (−33.5, 2.8)	0.237	0.09
55–64	16.8 (−39.2, 5.5)	0.104	0.14
≥65	51.7 (15.8, 87.5)	0.165	0.005

^a^ Model 1 is adjusted for age and sex. ^b^ Model 2 is adjusted for age, sex, white blood cell count, platelet count, body mass index, and presence of hypertension. 2 SD fasting blood glucose = 68.8 mg/dL. SII: systemic immune-inflammation index.

**Table 4:** Estimated mean difference in SII associated with 2 SD higher insulin and HbA1c, overall and by subgroups, *n* = 3895.

	SII	R^2^	*p* Value
	Mean Difference (95% CI)		
Insulin			
Model 1 ^a^	170.9 (−502.5, 844.3)	0.038	0.61
Model 2 ^b^	170.9 (−502.5, 844.3)	0.096	0.64
Subgroup analysis			
Female	173.2 (−401.3, 902.5)	0.095	0.64
Male	170.9 (−502.5, 844.3)	0.099	0.66
HbA1c			
Model 1 ^a^	18.9 (−16.1, 53.9)	0.004	0.19
Model 2 ^b^	30.6 (−51.9, 113.0)	0.023	0.28
Subgroup analysis			
Female	−434.3 (−1491.7, 623.2)	0.081	0.42
Male	−367.2 (−1560.2, 730.1)	0.062	0.45

^a^ Model 1 is adjusted for age and sex. ^b^ Model 2 is adjusted age, sex, white blood cell count, platelet count, BMI, and presence of hypertension. 2 SD insulin = 31.2 pmol/L. 2 SD HBA1C = 3.5%. HbA1c: hemoglobin A1c. SII: systemic immune-inflammation index.

## Data Availability

Data are available upon reasonable request.

## References

[1] International Diabetes Federation (2021). IDF Diabetes Atlas.

[2] Khan M.A.B., Hashim M.J., King J.K., Govender R.D., Mustafa H., Al Kaabi J. (2020). Epidemiology of type 2 diabetes—Global burden of disease and forecasted trends. J. Epidemiol. Glob. Health.

[3] Zhuo X., Zhang P., Barker L., Albright A., Thompson T.J., Gregg E. (2014). The lifetime cost of diabetes and its implications for diabetes prevention. Diabetes Care.

[4] Janssen L., Hiligsmann M., Elissen A., Joore M., Schaper N., Bosma J., Stehouwer C., Sep S., Koster A., Schram M. (2020). Burden of disease of type 2 diabetes mellitus: Cost of illness and quality of life estimated using the Maastricht Study. Diabet. Med..

[5] Harding J.L., Pavkov M.E., Magliano D.J., Shaw J.E., Gregg E.W. (2019). Global trends in diabetes complications: A review of current evidence. Diabetologia.

[6] Farmanfarma K.K., Ansari-Moghaddam A., Zareban I., Adineh H. (2020). Prevalence of type 2 diabetes in Middle–East: Systematic review& meta-analysis. Prim. Care Diabetes.

[7] Jarrar M., Abusalah M.A.H., Albaker W., Al-Bsheish M., Alsyouf A., Al-Mugheed K., Issa M.R., Alumran A. (2023). Prevalence of type 2 diabetes mellitus in the general population of Saudi Arabia, 2000–2020: A systematic review and meta-analysis of observational studies. Saudi J. Med. Med. Sci..

[8] Alwadeai K.S., Alhammad S.A. (2023). Prevalence of type 2 diabetes mellitus and related factors among the general adult population in Saudi Arabia between 2016–2022: A systematic review and meta-analysis of the cross-sectional studies. Medicine.

[9] Mokdad A.H., Tuffaha M., Hanlon M., El Bcheraoui C., Daoud F., Al Saeedi M., Alrasheedy A.A., Al Hussein M.A., Memish Z.A., Basulaiman M. (2015). Cost of diabetes in the Kingdom of Saudi Arabia, 2014. J. Diabetes Metab..

[10] Lontchi-Yimagou E., Sobngwi E., Matsha T.E., Kengne A.P. (2013). Diabetes mellitus and inflammation. Curr. Diabetes Rep..

[11] Hu B., Yang X.-R., Xu Y., Sun Y.-F., Sun C., Guo W., Zhang X., Wang W.-M., Qiu S.-J., Zhou J. (2014). Systemic immune-inflammation index predicts prognosis of patients after curative resection for hepatocellular carcinoma. Clin. Cancer Res..

[12] Xu J.-P., Zeng R.-X., Zhang Y.-Z., Lin S.-S., Tan J.-W., Zhu H.-Y., Mai X.-Y., Guo L.-H., Zhang M.-Z. (2023). Systemic inflammation markers and the prevalence of hypertension: A NHANES cross-sectional study. Hypertens. Res..

[13] Huang H., Liu Q., Zhu L., Zhang Y., Lu X., Wu Y., Liu L. (2019). Prognostic value of preoperative systemic immune-inflammation index in patients with cervical cancer. Sci. Rep..

[14] Yang R., Chang Q., Meng X., Gao N., Wang W. (2018). Prognostic value of systemic immune-inflammation index in cancer: A meta-analysis. J. Cancer.

[15] Li X., Gu L., Chen Y., Chong Y., Wang X., Guo P., He D. (2021). Systemic immune-inflammation index is a promising non-invasive biomarker for predicting the survival of urinary system cancers: A systematic review and meta-analysis. Ann. Med..

[16] Tian B.-W., Yang Y.-F., Yang C.-C., Yan L.-J., Ding Z.-N., Liu H., Xue J.-S., Dong Z.-R., Chen Z.-Q., Hong J.-G. (2022). Systemic immune–inflammation index predicts prognosis of cancer immunotherapy: Systemic review and meta-analysis. Immunotherapy.

[17] Ye Z., Hu T., Wang J., Xiao R., Liao X., Liu M., Sun Z. (2022). Systemic immune-inflammation index as a potential biomarker of cardiovascular diseases: A systematic review and meta-analysis. Front. Cardiovasc. Med..

[18] Li H., Huang J.-B., Pan W., Zhang C.-T., Chang X.-Y., Yang B. (2020). Systemic Immune-Inflammatory Index predicts prognosis of patients with COVID-19: A retrospective study. Res. Sq..

[19] Mangoni A.A., Zinellu A. (2023). Systemic inflammation index, disease severity, and mortality in patients with COVID-19: A systematic review and meta-analysis. Front. Immunol..

[20] Zhao Y., Shao W., Zhu Q., Zhang R., Sun T., Wang B., Hu X. (2023). Association between systemic immune-inflammation index and metabolic syndrome and its components: Results from the National Health and Nutrition Examination Survey 2011–2016. J. Transl. Med..

[21] Song Y., Guo W., Li Z., Guo D., Li Z., Li Y. (2022). Systemic immune-inflammation index is associated with hepatic steatosis: Evidence from NHANES 2015–2018. Front. Immunol..

[22] Nie Y., Zhou H., Wang J., Kan H. (2023). Association between systemic immune-inflammation index and diabetes: A population-based study from the NHANES. Front. Endocrinol..

[23] Liu P., Shang J., Luo D., Shi L. (2023). The systemic immune-inflammation index is associated with Type 2 diabetes mellitus patients: Evidence from NHANES 2011–2018. Res. Sq..

[24] Mann C. (2003). Observational research methods. Research design II: Cohort, cross sectional, and case-control studies. Emerg. Med. J..

[25] Flack J.M., Adekola B. (2020). Blood pressure and the new ACC/AHA hypertension guidelines. Trends Cardiovasc. Med..

[26] (2022). General Authority of Statistics 2022 Census Report. General Authority of Statistics, Kingdom of Saudi Arabia. https://portal.saudicensus.sa/portal/public/1/15.

[27] Yang C., Yang Q., Xie Z., Peng X., Liu H., Xie C. (2023). Association of systemic immune-inflammation-index with all-cause and cause-specific mortality among type 2 diabetes: A cohort study base on population. Endocrine.

[28] Elbeyli A., Kurtul B.E., Ozcan S.C., Ozarslan Ozcan D. (2022). The diagnostic value of systemic immune-inflammation index in diabetic macular oedema. Clin. Exp. Optom..

[29] Guo W., Song Y., Sun Y., Du H., Cai Y., You Q., Fu H., Shao L. (2022). Systemic immune-inflammation index is associated with diabetic kidney disease in Type 2 diabetes mellitus patients: Evidence from NHANES 2011–2018. Front. Endocrinol..

[30] Ozer Balin S., Ozcan E.C., Uğur K. (2022). A new inflammatory marker of clinical and diagnostic importance in diabetic foot infection: Systemic immune-inflammation index. Int. J. Low. Extrem. Wounds.

[31] Wada J., Makino H. (2016). Innate immunity in diabetes and diabetic nephropathy. Nat. Rev. Nephrol..

[32] Oertelt-Prigione S. (2012). The influence of sex and gender on the immune response. Autoimmun. Rev..

[33] Meng X., Chang Q., Liu Y., Chen L., Wei G., Yang J., Zheng P., He F., Wang W., Ming L. (2018). Determinant roles of gender and age on SII, PLR, NLR, LMR and MLR and their reference intervals defining in Henan, China: A posteriori and big-data-based. J. Clin. Lab. Anal..

[34] Grossmann V., Schmitt V.H., Zeller T., Panova-Noeva M., Schulz A., Laubert-Reh D., Juenger C., Schnabel R.B., Abt T.G., Laskowski R. (2015). Profile of the immune and inflammatory response in individuals with prediabetes and type 2 diabetes. Diabetes Care.

[35] Harris M.I., Eastman R.C. (2000). Early detection of undiagnosed diabetes mellitus: A US perspective. Diabetes/Metab. Res. Rev..

[36] Duncan B.B., Schmidt M.I., Pankow J.S., Ballantyne C.M., Couper D., Vigo A., Hoogeveen R., Folsom A.R., Heiss G. (2003). Low-grade systemic inflammation and the development of type 2 diabetes: The atherosclerosis risk in communities study. Diabetes.

[37] Bertoni A.G., Burke G.L., Owusu J.A., Carnethon M.R., Vaidya D., Barr R.G., Jenny N.S., Ouyang P., Rotter J.I. (2010). Inflammation and the incidence of type 2 diabetes: The Multi-Ethnic Study of Atherosclerosis (MESA). Diabetes Care.

[38] Samer H., Wessam G., Ibrahim J., Barría R.M. (2018). Prospective Cohort Studies in Medical Research. Cohort Studies in Health Sciences.

[39] Islam M.M., Satici M.O., Eroglu S.E. (2024). Unraveling the clinical significance and prognostic value of the neutrophil-to-lymphocyte ratio, platelet-to-lymphocyte ratio, systemic immune-inflammation index, systemic inflammation response index, and delta neutrophil index: An extensive literature review. Turk. J. Emerg. Med..

[40] Bauhoff S. (2014). Self-report bias in estimating cross-sectional and treatment effects. Encyclopedia of Quality of Life and Well-Being Research.

[41] Hu F.B. (2011). Globalization of diabetes: The role of diet, lifestyle, and genes. Diabetes Care.

